# MIST, a Novel Approach to Reveal Hidden Substrate Specificity in Aminoacyl-tRNA Synthetases

**DOI:** 10.1371/journal.pone.0130042

**Published:** 2015-06-11

**Authors:** Gilbert Eriani, Joseph Karam, Jomel Jacinto, Erin Morris Richard, Renaud Geslain

**Affiliations:** 1 Architecture et Réactivité de l'ARN, Université de Strasbourg, CNRS, Institut de Biologie Moléculaire et Cellulaire, 67084, Strasbourg, CEDEX, France; 2 Laboratory of tRNA Biology, Department of Biology, College of Charleston, Charleston, South Carolina, United States of America; Institute of Molecular Genetics IMG-CNR, ITALY

## Abstract

Aminoacyl-tRNA synthetases (AARSs) constitute a family of RNA-binding proteins, that participate in the translation of the genetic code, by covalently linking amino acids to appropriate tRNAs. Due to their fundamental importance for cell life, AARSs are likely to be one of the most ancient families of enzymes and have therefore been characterized extensively. Paradoxically, little is known about their capacity to discriminate tRNAs mainly because of the practical challenges that represent precise and systematic tRNA identification. This work describes a new technical and conceptual approach named MIST (Microarray Identification of Shifted tRNAs) designed to study the formation of tRNA/AARS complexes independently from the aminoacylation reaction. MIST combines electrophoretic mobility shift assays with microarray analyses. Although MIST is a non-cellular assay, it fully integrates the notion of tRNA competition. In this study we focus on yeast cytoplasmic Arginyl-tRNA synthetase (yArgRS) and investigate in depth its ability to discriminate cellular tRNAs. We report that yArgRS in submicromolar concentrations binds cognate and non-cognate tRNAs with a wide range of apparent affinities. In particular, we demonstrate that yArgRS binds preferentially to type II tRNAs but does not support their misaminoacylation. Our results reveal important new trends in tRNA/AARS complex formation and potential deep physiological implications.

## Introduction

Aminoacyl-tRNA synthetases (AARSs) constitute a family of RNA-binding proteins that participate in the translation of the genetic code by covalently linking amino acids to the 3’ end of appropriate tRNAs [1]. AARSs are divided into two classes, of 10 members each, based on structural and sequence features of their active sites [2, 3]. They aminoacylate tRNA in two steps: amino acids are first activated at the expense of ATP followed by the transfer of the amino acid moieties onto tRNAs. Some AARSs such as Arginyl-tRNA synthetases (ArgRS) require the cognate tRNA to synthesize aminoacyl-AMP in the first step [4].

tRNAs are short non-coding RNA displaying an “L-shaped” three-dimensional structure. Amino acids are transferred onto the acceptor extremity whereas the other end base pairs with codons on the messenger RNA. These two tRNA-extremities are directly involved in complex formation while the contribution of the core is typically low [5]. Class I and class II AARSs approach their tRNAs from opposite sides forming complexes that are mirror images of each other [6].

In order to interact uniformly with the cellular translation machinery, tRNAs display similar secondary and tertiary structures. Because tRNAs look alike, AARSs are thought to poorly discriminate tRNAs at the binding step; consequently accurate aminoacylation relies essentially on kinetic specificity [7]. In this widely accepted model, tRNA/AARS complexes form indifferently; homologous complexes between tRNA and AARS of the same specificity largely outcompete heterologous complexes preferentially yielding properly aminoacylated tRNA. To enable efficient kinetic discrimination, tRNAs display a combination of positive and negative signals called determinants [8]. Positive determinants promote the transfer of cognate amino acids by the corresponding AARSs whereas negative determinants prevent or limit misaminoacylation.

As technology evolves, detection of misaminoacylation improves. An increasing body of evidence demonstrates that misaminoacylation of tRNA by AARS is present throughout the three domains of life. For example, tRNA mischarging is a necessity in organisms lacking AsnRS and/or GlnRS [9, 10]. In *Mycoplasma* parasites several atypical AARSs catalyze high levels of tRNA mischarging resulting in statistical proteomes [11]. Fungi of the genus *Candida* misaminoacylate tRNA Leu and rely on ambiguous decoding to increase phenotype diversity [12]. MetRSs misaminoacylate various tRNAs in Humans in response to oxidative stress [13]. In *Mycoplasma capricolum*, transplanted pyrrolysine tRNA is aminoacylated by ArgRS [14].

The fact that some AARSs bind and aminoacylate non-cognate tRNAs at a substantial rate *in vivo* challenges the common belief that AARSs bind preferentially to cognate over non-cognate tRNAs. Do all cellular tRNAs really uniformly bind to a given AARS? Or is overall aminoacylation the combined action of differential tRNA binding and kinetic specificities? If so, what are the differences in affinity and what are the mechanistic implications?

We chose Saccharomyces cerevisiae Arginyl-tRNA synthetase (yArgRS), as a model to evaluate tRNA binding discrimination, for the following reasons. The structures and functions of yArgRS have been extensively characterized. In addition, yArgRS misaminoacylates tRNA Asp *in vitro* at a significant rate. Previous structural, genetic and *in vitro* based studies have shown that yArgRS interacts with tRNA Arg in a standard class I fashion. The enzyme contacts the tRNA on the D loop side and interacts with the major groove of the acceptor stem as well as the anticodon loop. Arginine determinants are C35 and G36 or U36. [15–17].

The *S*. *cerevisiae* nuclear genome encodes 77 different tRNA genes organized in 42 tRNA isoacceptors. Yeast tRNA display 25 different nucleoside modifications that are added post-transcriptionally. Nearly 15% of yeast tRNA residues are nucleosides other than A, G, U, or C [18]. These modified nucleosides serve numerous important functions including tRNA discrimination, translation fidelity, and tRNA quality control [19].

*In vitro* aminoacylation and gel retardation assays are extremely valuable tools to estimate the dissociation constant of AARS complexed to individual tRNA species. However, none of these approaches allow non-biased and systematic assessment of tRNA specificity at the binding step. In this study we decoupled binding from aminoacylation to study the early step of complex formation. We developed an original method where complexed tRNAs isolated from total tRNA extracts through electrophoretic mobility shift assays were streamlined to tRNA microarrays for systematic identification. We named this approach MIST for Microarray Identification of Shifted tRNA. We report here that in submicromolar concentrations, yArgRS binds cognate and non-cognate tRNAs with a wide range of apparent affinities. In particular, yArgRS interacts preferentially with non-cognate class 2 tRNA Ser and Leu. We discuss the mechanistic and potential physiological implications of these findings.

## Methods

### Purification of yeast ArgRS

yArgRS was expressed as a native recombinant protein, free of purification tag, from pTrc99B plasmid in the *E*. *coli* strain TB1 (F—ara D (lac-proAB) hsdR (rk—mk+) rpsL (Strr); [f80, dlacD (lacZ) M15]) based on a protocol initially established for yeast Aspartyl-tRNA synthetase [20]. The co-expression of a plasmidic copy of the *E*. *coli* tRNA Arg (UCU) gene enhanced the translation of AGG and AGA codons poorly used in bacteria but inversely represented in yeast genes. The double-transformed TB1 was grown in 100 ml of LB to A_700_ nm = 0.5 and induced by 0.5 mM IPTG for 12 h. Cells were washed with TE buffer (10 mM Tris—HCl, pH 8.0, 1 mM EDTA), resuspended in 8 ml of 100 mM Tris—HCl, pH 8.0, 10 mM MgCl_2_ and 1 mM EDTA and submitted to 5 cycles of sonication on ice (20 s each at 120 V, Annemasse Ultrasons sonifier 250TS20K). The supernatant of a 150 min-centrifugation at 105,000 x g was subjected to three chromatographic column separations on DEAE Sephacel (GE Healthcare), HA Ultrogel (Sigma Aldrich) and reverse ammonium sulfate on TSK HW65 (S) (Merck). Purified yArgRS was dialyzed overnight at 4°C in 50 mM Tris-HCl, pH 7.5, 1 mM MgCl_2_, 0.1 mM EDTA and glycerol 60% (v/v). One hundred ml of recombinant TB1 yielded 2.5 mg of yArgRS, pure at 99.9%. The charging activity of the purified yArgRS was tested in the following conditions: 100 mM HEPES-KOH (pH 7.5), 30 mM KCl, 0.1 mg/mL bovine serum albumin, 5 mM glutathione, 5 mM ATP, 50 μCi L-[^14^C] arginine (313 Ci/mol), 5 mM MgCl_2_, and 10 μM of native yeast tRNA Arg (ICG). The catalytic activity of the purified yArgRS is 8 s^-1^.

### Preparation of total tRNAs for the EMSA

Total RNAs were Trizol extracted from *S*. *cerevisiae* cultures grown in the exponential phase. The RNA extract was separated on a 10% denaturing PAGE containing 7 M urea. The band corresponding to total tRNAs was visualized by UV shadowing and tRNAs were extracted by soaking gel slices for 1 hour at 50°C in 50 mM KOAc and 200 mM KCl, pH 7. Purified total tRNAs were then deacylated 30 min at 37°C in 1 M Tris—HCl, pH 8.8, neutralized, precipitated and resuspended in H_2_O.

### Radiolabeling of total tRNAs

Two μg of total tRNAs were dephosphorylated for 30 min at 37°C with 1 unit of calf-intestine alkaline phosphatase. Dephosphorylated tRNAs were radiolabeled in 5’ for 30 min at 37°C with 5 units of T4 polynucleotide kinase and 25 pmol of 6000 Ci/mmol [γ-^32^P] ATP. Labeled tRNAs were gel purified as described above.

### Electrophoretic mobility shift assay

5’-labeled total tRNAs (> 10^5^ cpm) were incubated with variable concentrations of purified yArgRS (0.2 to 29.0 μM) for 15 min at room temperature. Complexes were formed in 10 μl of binding buffer containing 25 mM Tris-HCl, pH 7.5, 75 mM KCl, 10 mM MgCl_2_, 25% glycerol and separated at room temperature for 120 min at 120V on native 6% (37.5:1) acrylamide:bisacrylamide gels (200 × 200 × 1.5 mm^3^). The binding buffer for the experiment described in S1 Fig. was complemented with β,γ-methylene adenosine 5′-triphosphate (a non-hydrolysable analog of ATP) 10 mM and arginine 50 μM.

### tRNA microarray

The standard tRNA microarray experiment consists of 3 steps starting from manual printing, followed by hybridization and finally data analysis.

The microarray printing conditions were the same as those described by Dittmar and collaborators [21]. The nuclear genome of *S*. *cerevisiae* encodes a total of 77 different tRNA Arg sequences. 48 non- or partially-degenerated DNA oligonucleotide probes were designed on the basis of the 2011 *S*. *cerevisiae* tRNA gene annotation. Eight replicates of each probe were printed on each array. The sequences of the DNA oligonucleotide probes are provided in S1 File.

Hybridization of 5’ labeled tRNA was performed at 60°C for 4 hours and followed by several wash steps of decreasing stringencies [21].

Arrays were scanned at 50μm resolution using a GE Healthcare storm 865 scanner. Radioactivity intensities at each probe spot were quantified with Image J software (microarray profiler). Heat maps were generated on Microsoft Excel using the conditional formatting tool.

### tRNA *in vitro* transcription

tRNAs were transcribed *in vitro* from a template generated by primer extension of overlapping DNA oligonucleotides (IDT) in 250 μl of 200 mM Tris-HCl, pH 8, 100 mM spermidine, 1 mg BSA, 250 mM MgCl_2_, 250 mM DTT and 15,000 units of T7 RNA polymerase (Lucigen) [22]. Transcripts were then purified on 10% denaturing PAGE as described above.

### Measure of tRNA aminoacylation levels *in vivo*

Extraction of RNA under acidic conditions and acid gel separation allows the measurement of the aminoacylation levels of tRNAs in vivo [23]. Acidic conditions preserve the ester bond between the tRNA and the amino acid, therefore aminoacylated tRNA can be separated from uncharged tRNA and visualized by hybridization with a specific probe.

Yeast cells were grown in 100 ml of complete YPD medium to A700 nm = 2 and harvested by centrifugation. All subsequent steps were performed on ice. The cell pellet was resuspended in 5 ml of extraction buffer (0.3 M sodium acetate, pH 4.5, 10 mM EDTA) and subjected to two acid-phenol extractions (pH 4.5). Thirty μg of extracted total tRNAs were separated on a 1 mm thick 6.5% polyacrylamide gel (19:1 acrylamide:bisacrylamide) containing 8 M urea in 0.1 M sodium acetate buffer pH 5. Electrophoresis was performed at 18 W (400 V) at 4°C for 24 h.

Separated RNAs were transferred from the gel onto a nylon membrane (Hybond-XL) and cross-linked at 80°C using a gel dryer. Pre-hybridization was performed for 4 h at 60°C in 40 ml of 1× Denhardt’s solution, 5× SSPE, 0.5% SDS (w/v). Hybridization was performed for 12 h at 60°C in 15 ml of the same solution, in the presence of 5′ 32P-labeled DNA probes (20-mers) specific to the 3’ end of tRNA Arg (CCG) and a tRNA Leu (UAA) [17]. Washing was performed in 2× SSPE and 0.5% SDS for 20 min at 50°C. Radioactive signal was visualized using a GE Healthcare storm 865 scanner.

## Results

### Isolation of complexed tRNA by EMSA

**Fig 1:**
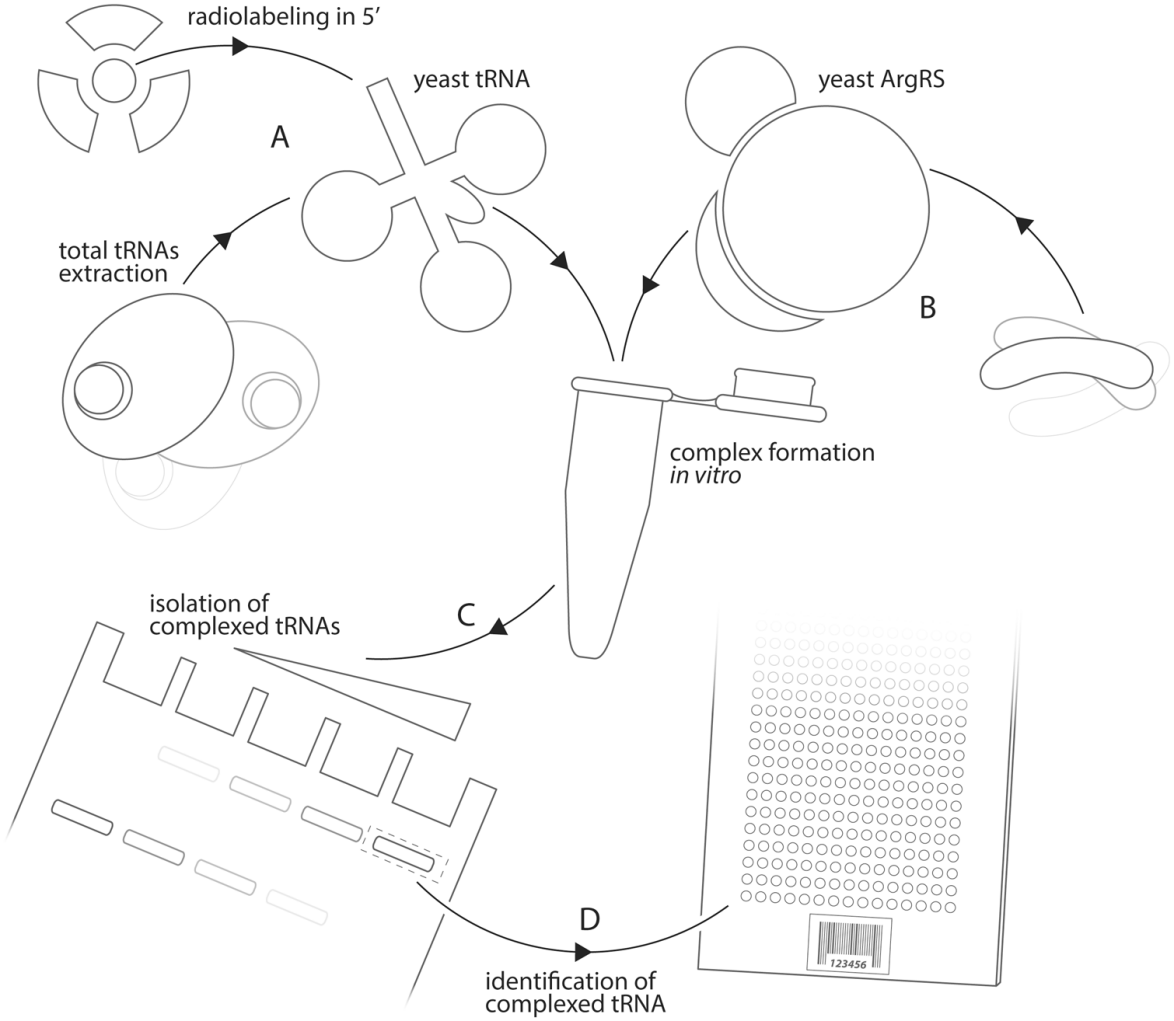
Schematic description of the method used for the identification of tRNA complexed by yArgRS. (A) Yeast total tRNAs were prepared by deacylation and gel purification of a yeast extract of total RNA and subsequently radiolabeled in 5’ with ^32^P. (B) Yeast Arginyl-tRNA synthetase (yArgRS) was expressed as a recombinant protein in *E*. *coli* and purified by FPLC. (C) Fixed amounts of labeled tRNA were incubated 15 min on ice with varying amounts of purified enzyme ranging from 0.2 to 29 μM. Free tRNAs were separated from complexed species on non-denaturing gel. (D) Complexed tRNAs corresponding to high molecular weight bands were eluted and hybridized on tRNA microarrays.

tRNAs bind to aminoacyl-tRNA synthetases through a complex and dense network of non-covalent interactions. Both the strength and specificity of this interaction network are affected by factors such as the presence of bulky molecular labels, post-transcriptional modifications, as well as the relative concentration of each tRNA species in the sample. In order to best mimic physiological conditions, we obtained fully modified total tRNA from a yeast culture grown under optimal conditions. These tRNA were deacylated and labeled by exchanging their 5’P with ^32^P (Fig 1). A tRNA sample prepared accordingly remains fully functional in translation and preserves its stoichiometry [22].

**Fig 2:**
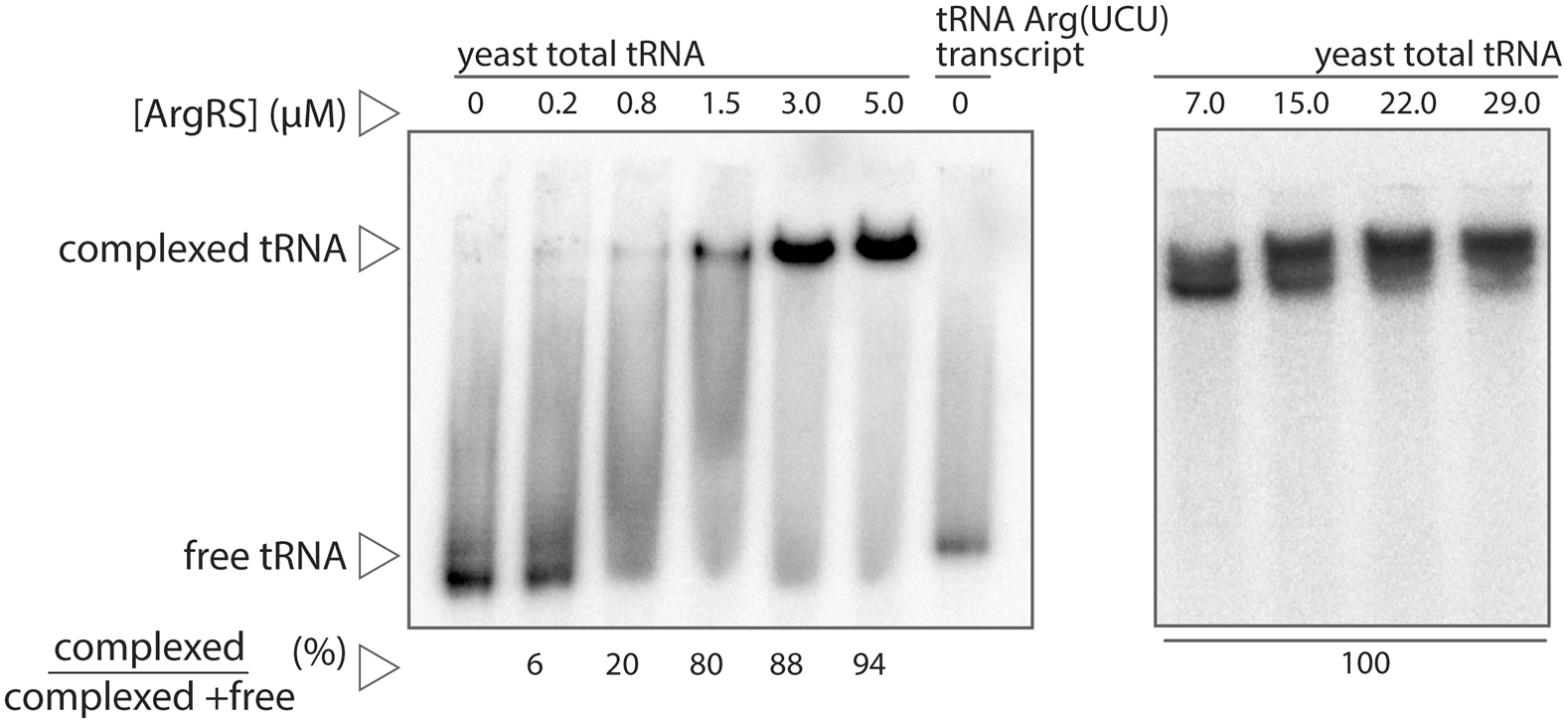
Separation of free and complexed tRNAs by EMSA. Radiolabeled yeast total tRNAs and in vitro transcribed yeast tRNA Arg (UCU) (as a migration control) were incubated with 0 to 29 μM of yArgRS and separated on a 6% non-denaturing polyacrylamide gel. The fraction of shifted or complexed tRNAs is indicated.

Complexes between labeled tRNAs and purified yArgRS were formed *in vitro* and separated from free tRNA species by non-denaturing gel electrophoresis. The principal limitation to this approach for the subsequent microarray analysis is signal intensity. We observed that the proportion of complexed tRNA increased gradually with enzyme concentrations (Fig 2) reaching nearly 95% at 5 μM. Complexes prepared with as low as 0.8 μM yielded workable amounts of tRNA for microarray analysis. A second complex of higher molecular weight gradually formed beyond 5 μM. At high concentrations, yArgRS was previously reported to spontaneously dimerize in the presence of tRNA [24]. Constrained by signal intensity and the enzyme’s inclination toward oligomerization, we restricted our analysis to the 0.8 and 5 μM range. Interestingly, a vast majority of tRNAs were complexed at low enzyme concentration (3 and 5 μM), suggesting that yArgRS not only interacts with tRNA Arg species but also binds readily to non-cognate tRNAs.

To evaluate if small yArgRS substrates such as ATP and arginine could modify the migration pattern on EMSA, we repeated the experiment under the same conditions with the addition, this time, of β,γ-methylene adenosine 5′-triphosphate 10 mM (a non-hydrolysable analog of ATP) and L-arginine 50 μM in the binding mixture (prior to gel separation). These concentrations mimic the optimal ATP and arginine concentrations established for *in vitro* aminoacylation assays. No significant differences were observed for the two conditions (with and without small substrates), which suggest that the small substrates do not significantly influence tRNA binding specificity (S1 Fig).

### MIST: microarray identification of shifted tRNAs—a proof of concept

The main challenges with tRNA identification in a mixture are both the substantial number of different molecules as well as their close resemblance [25]. tRNAs have comparable biophysical properties, parameters such as volume or charge are nearly identical which complicates systematic separation by chromatography. However, most tRNAs display great variation in their primary sequences, making identification through selective hybridization to a complementary probe a method of choice [26]. The nuclear genome of *S*. *cerevisiae* encodes a total of 287 tRNA genes, which represents 77 unique tRNA sequences organized in 42 different tRNA isoacceptors families (tRNAs with different anticodons) [27]. Seven percent of the yeast tRNA gene population encode arginine decoding tRNA. Less than half of yeast’s tRNA pool consists of isodecoders, tRNAs that share the same anticodon but have slightly different body sequences. As a comparison, the human genome encodes nearly 90% of tRNA isodecoders [25]. Isodecoders are virtually indistinguishable through standard hybridization methods [28]. To enable homogenous binding some DNA probes were degenerated at a maximum 5 positions. Arrays prepared manually consist in 8 spots or replicas per probe for a total of 384 probes. The binding capacity of each array is in the mid nanogram range.

In standard microarray analysis both sample and reference are loaded together on the same array. They are typically labeled with different fluorophores and yield non-overlapping signals that are separately quantifiable. Fluorophores are typically bulky polycyclic molecules. Although they are widely used in RNA labeling they are often unsuitable to study tRNA-protein interactions because of potential steric hindrance. Variations in array hybridization and gel extraction efficiencies potentially bias the analysis. Therefore, array-to-array comparison cannot be performed directly and requires normalization. As a consequence we normalized the signal intensity for each tRNA to the signal of all spots, where each tRNA signal is represented as a fraction of all signals combined. This allows an unequivocal comparison of relative tRNA concentrations in each sample. For rapid visual evaluation, results were displayed as heat maps.

**Fig 3:**
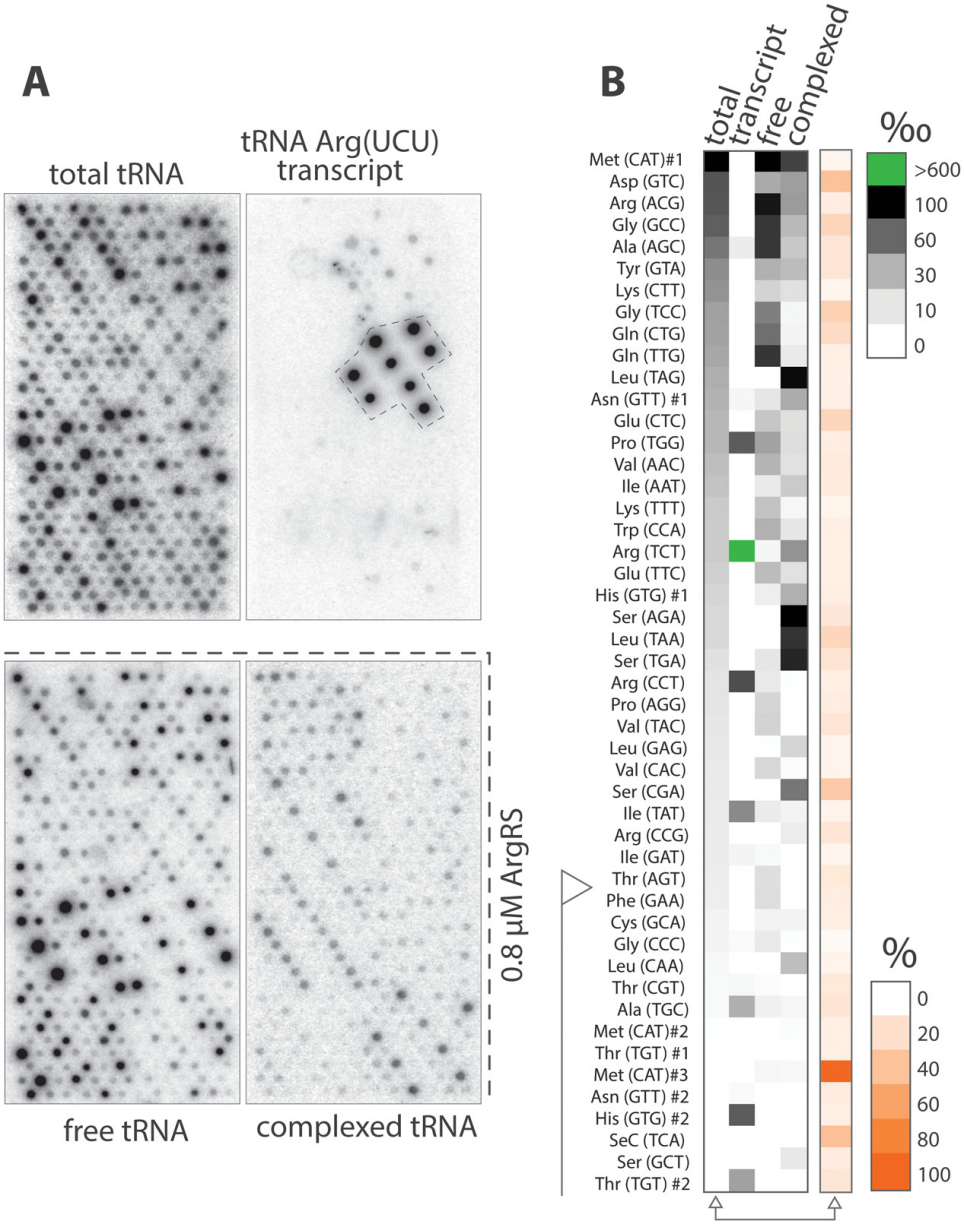
Microarray analysis of complexed tRNAs. (A) From top to bottom, in house printed tRNA microarrays hybridized with yeast radiolabeled: (i) total tRNA, (ii) *in vitro* transcribed tRNA Arg (UCU) (as a cross-hybridization control), (iii) free and (iv) complexed tRNAs after separation in the presence of 0.8 μM yArgRS and gel elution. Each array displays 384 spots corresponding to 48 probes replicated 8 times. (B) Radioactive signal of the four above-mentioned tRNA samples hybridized on the arrays. Relative signal intensity for each probe corresponds to the intensity of the probe (median of 8 spots) divided by the total intensity on the array expressed in per mille. Relative intensities are represented as shades of grey on the heat map. The probes are organized from high to low relative intensity in the total tRNA sample. The flag indicates the probes that were not considered for further analysis due to low signal intensity. Standard deviations of the 8 replicas for each probe in the total tRNA sample are indicated as shades of red.

Post-transcriptional modifications and subtle variations in tRNA structures in or near the acceptor stem can potentially influence 5’ labeling by the polynucleotide kinase [29]. As anticipated, 5’ labeling of total tRNA is highly heterogeneous (Fig 3, S2 Fig and S2 File). The top 5 probes in intensity represent nearly 40% of the signal on the array (Fig 3, first column). 14 probes gave signals near or below the detection threshold and were not considered for further analysis (from Phe (GAA) to the bottom). This distribution does not reflect the relative concentration of each tRNA in the sample.

Sound microarray-based approaches typically minimize cross-hybridization and rely on signal consistency for replicas of the same probe. The reproducibility of the tRNA array method and result validation by Northern blot have been extensively described [21, 26]. A partial quality assessment was nevertheless conducted on one hand using a radiolabeled tRNA Arg (UCU) transcript. It revealed that less than 30% of the total signal resulted in off-target binding even in the absence of competition (Fig 3, second column). On the other hand 39 probes out of 48 displayed a standard deviation equal to or below 17% (Fig 3, fifth column).

Surprisingly, microarrays revealed that free and complexed tRNAs at 0.8 μM (Fig 3, third and fourth columns) were clearly distinct populations. In the sub-micromolar range, yArgRS binds preferentially non-cognate tRNA.

### Genome wide exploration of tRNA binding specificity

**Fig 4:**
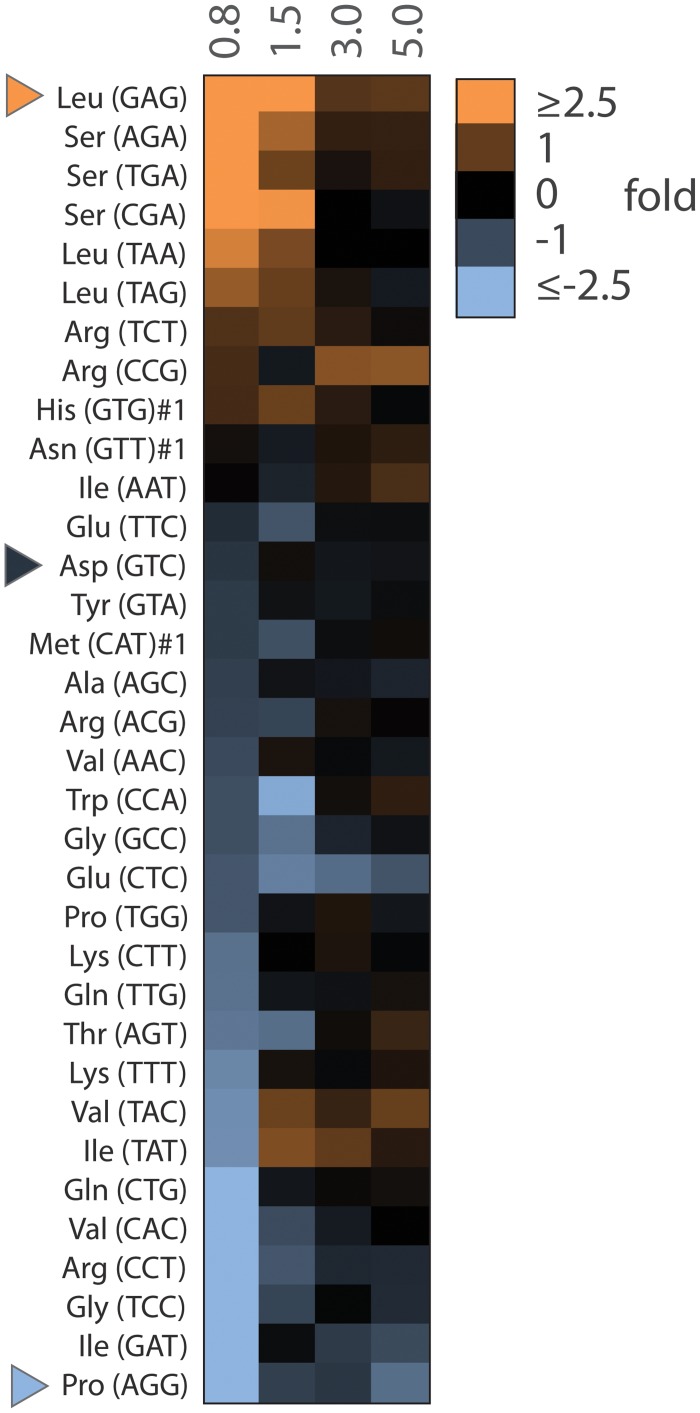
Relative abundance of complexed tRNAs. Data is shown for four enzyme concentrations all relative to total tRNAs. In other words, it corresponds for each probe, to the relative signal intensity in the complexed tRNA sample divided by the relative signal intensity in total tRNAs (EMSA input), expressed as fold differences. Red indicates an increase in relative amount whereas blue indicates a decrease. Data is organized from high to low relative abundance at 0.8 μM.

**Fig 5:**
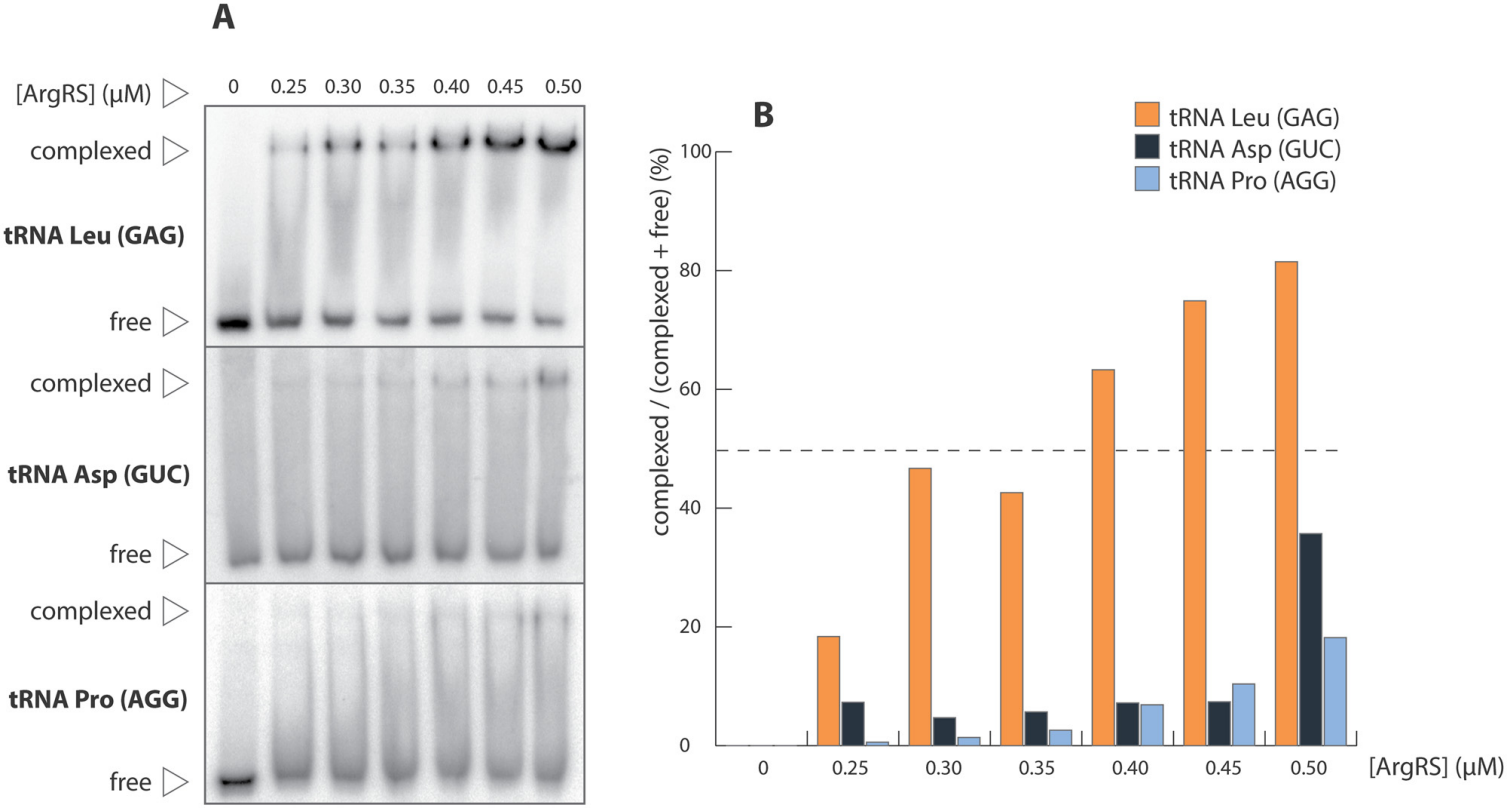
Complexation of yArgRS with isolated tRNAs. (A) Radiolabeled, *in vitro* transcribed, tRNA Leu (GAG), Asp (GUC) and Pro (AGG) displaying respectively high, medium and low apparent affinities under competition were tested individually in the presence of various concentrations of yArgRS ranging from 0.25 to 0.50 μM on non-denaturing PAGE. (B) Fractions of complexed tRNA at different enzyme concentrations. The color code corresponds to the relative abundance of each tRNA species at 0.8 μM (Fig 4, first column).

tRNAs complexed in the presence of 0.8, 1.5, 3.0 and 5.0 μM of yArgRS were recovered by gel elution and hybridized on microarrays. In order to evaluate their ability to bind to yArgRS, the relative amounts (RA) of each tRNA species in the four samples were normalized to the corresponding relative amounts in total tRNA (input) (Fig 4). In other words for any given tRNA the loss or gain in RA was expressed as: log 2 (RA sample / RA input). In Fig 4, red on the heat map indicates enrichment compared to total tRNA; conversely blue indicates depletion. At 0.8 μM, two tRNA species displayed a notable gain in RA (>3 fold): tRNA Leu (GAG) and Ser (AGA). tRNA Arg (CCT), Gly (TCC), Ile (GAT) and Pro (AGG) displayed a notable loss (<0.3 fold). As expected, RA ratios level off at higher enzyme concentrations as most tRNAs are found in their complexed form. We chose 3 tRNA species with apparent high (tRNA Leu (GAG)), medium (tRNA Asp (GTC)) and low (tRNA Pro (AGG)) affinity for yArgRS at 0.8 μM for further individual characterization. The corresponding transcripts were incubated individually in the presence of various concentrations of yArgRS ranging from 0.25 to 0.50 μM. At 0.5 μM over 60% of Leucine transcripts were in their complexed form as opposed to 35% and 19% for Asp and Pro respectively, supporting the microarray observations (Fig 5). Even in the absence of competition, tRNA Leu stripped of all post-transcriptional modifications remained the dominant binding species suggesting the presence of a strong binding attribute in its primary sequence.

### tRNA size matters

Despite sharing a conserved three-dimensional structure, cellular tRNAs vary vastly in intracellular abundance and primary sequence. These features influence tRNA-AARS interactions to a great extent. In lower eukaryotes, the number of gene copies encoding tRNAs is commonly used as a proxy for the relative intracellular tRNA concentrations. The analysis of apparent affinities versus predicted concentrations did not reveal any obvious relationship between the two variables in our data set (R^2^ = 0.027, data not shown). For example, tRNA Gly (GCC) with 16 gene copies has a 4.5 fold lower apparent affinity compared to tRNA Leu (GAG) with one gene copy.

**Fig 6:**
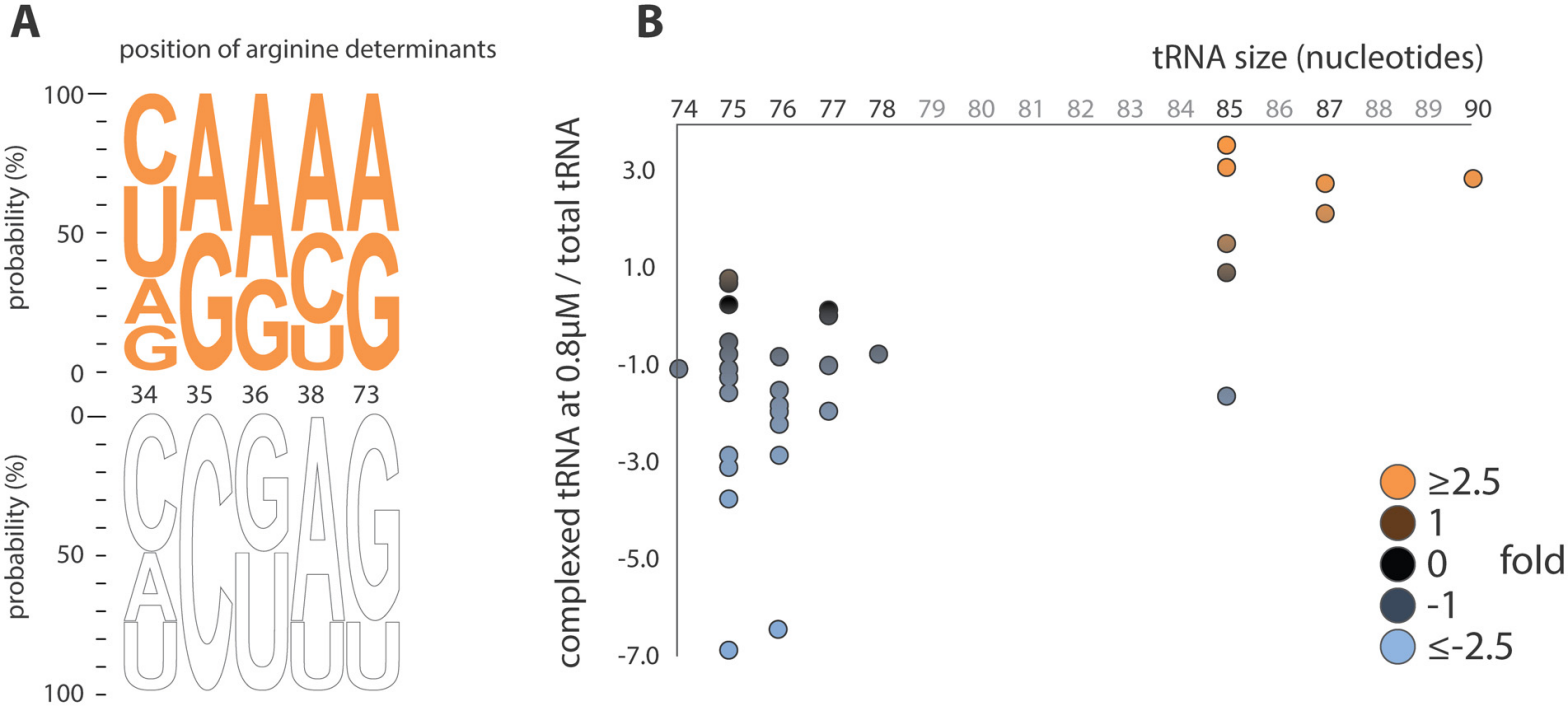
tRNA features versus apparent affinity for yArgRS. (A) Sequence logo comparing the identity elements in yeast tRNA Arg (white, outlined letters) with the nucleotides at the corresponding positions in non-cognate tRNA species displaying apparent high affinity for yArgRS (top 6 tRNAs at 0.8 μM in Fig 4). (B) Association of tRNA sizes to apparent affinity for yArgRS. The color code corresponds to the relative abundance of each tRNA species at 0.8 μM (Fig 4, first column).

yArgRS aminoacylates and consequently binds cognate and non-cognate tRNAs displaying proper identity elements in the anticodon loop (34, 35, 36 and 38) and the discriminator base (73) [15–17]. However, none of the species with the highest apparent affinity for yArgRS presented clear aminoacylation determinants suggesting the existence of an independent set of binding determinants (Fig 6 and S3 File).

We detected significant differences between two size groups (71–75 nucleotides and 82–87 nucleotides) in binding ability (F-23.39, df = 37, p<0.0001) with analysis of a variance model (PROC GLM in SAS, version 9.3). Class 2 tRNAs are characterized by the presence of an extended variable loop which provides substantial binding energy for the formation of the corresponding tRNA-AARS complex [30, 31]. tRNA Leu and Ser are all class 2 in yeast. Surprisingly, all six class 2 species ranked in the top 6 for tRNAs with the highest apparent affinity for yArgRS (Fig 6) demonstrating that yArgRS binds preferentially to class 2 tRNAs.

### Catalytic specificity offsets apparent poor binding discrimination

tRNA aminoacylation is the product of a two-pronged reaction starting with the formation of a tRNA-AARS complex followed by the transfer of an amino acid onto the tRNA 3’ end. yArgRS, as any other RNA binding proteins, displays an RNA binding area with compatible topography and electrostatic potential [16]. As tRNAs share similar 3D structures they are typically poorly discriminated at the binding step. The core mechanism for cognate tRNA aminoacylation relies on the proper positioning of the acceptor stem of complexed tRNA in the enzyme active site.

The aminoacylation specificities of tRNA Leu (UAA) and control tRNA Arg (CCG) were estimated by northern blot under acidic conditions to limit the hydrolysis of the aminoacyl-tRNA ester bonds. More specifically, total RNAs were extracted from a yeast culture and separated on denaturing PAGE before being transferred onto a nylon membrane. The membrane was hybridized with a mixture of ^32^P-labeled oligonucleotides complementary to tRNA Leu (UAA) and tRNA Arg (CCG).

**Fig 7:**
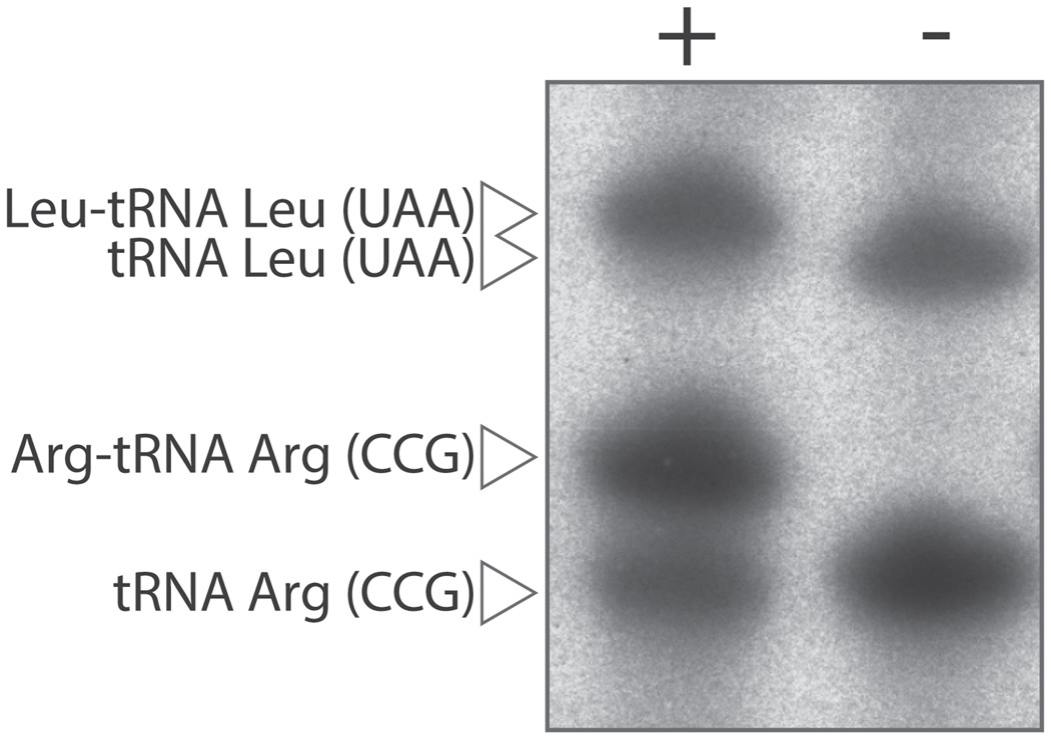
Northern blot analysis of yeast total RNAs under acidic conditions. Total RNAs were extracted and separated under acidic conditions before being transferred onto a nylon membrane. The membrane was probed with a mixture of ^32^P-labeled oligonucleotides complementary to tRNA Leu (UAA) and tRNA Arg (CCG). Acylated (+) and deacylated (-) tRNAs were loaded on separate lanes. The shift induced by the presence of arginine residues is significantly greater than the one induced by leucine. *In vivo*, tRNA Leu (UAA) is not significantly arginylated despite a strong affinity for yArgRS.

The gel mobility of aminoacyl-tRNAs depends both on the size and the charge of their amino acids. Leucine and arginine have comparable molecular weight (131 and 174 g/mol respectively) but different charges. With a pKa of 12.5, arginine is positively charged in acidic environments whereas leucine is electrically neutral. As a consequence, tRNA species simultaneously aminoacylated with arginine and leucine should yield two distinct bands by northern blot. As anticipated, we observed that the shift induced by arginine residues is significantly larger compared to leucine. On the northern blot, aminoacylated tRNA Leu migrate as a single spot demonstrating that misarginylation does not occur at a significant level (Fig 7). Despite a superior binding ability, non-cognate tRNA Leu (UAA) are not significantly aminoacylated by yArgRS *in vivo*.

### ArgRS displays disparate affinity for cognate tRNAs

**Fig 8:**
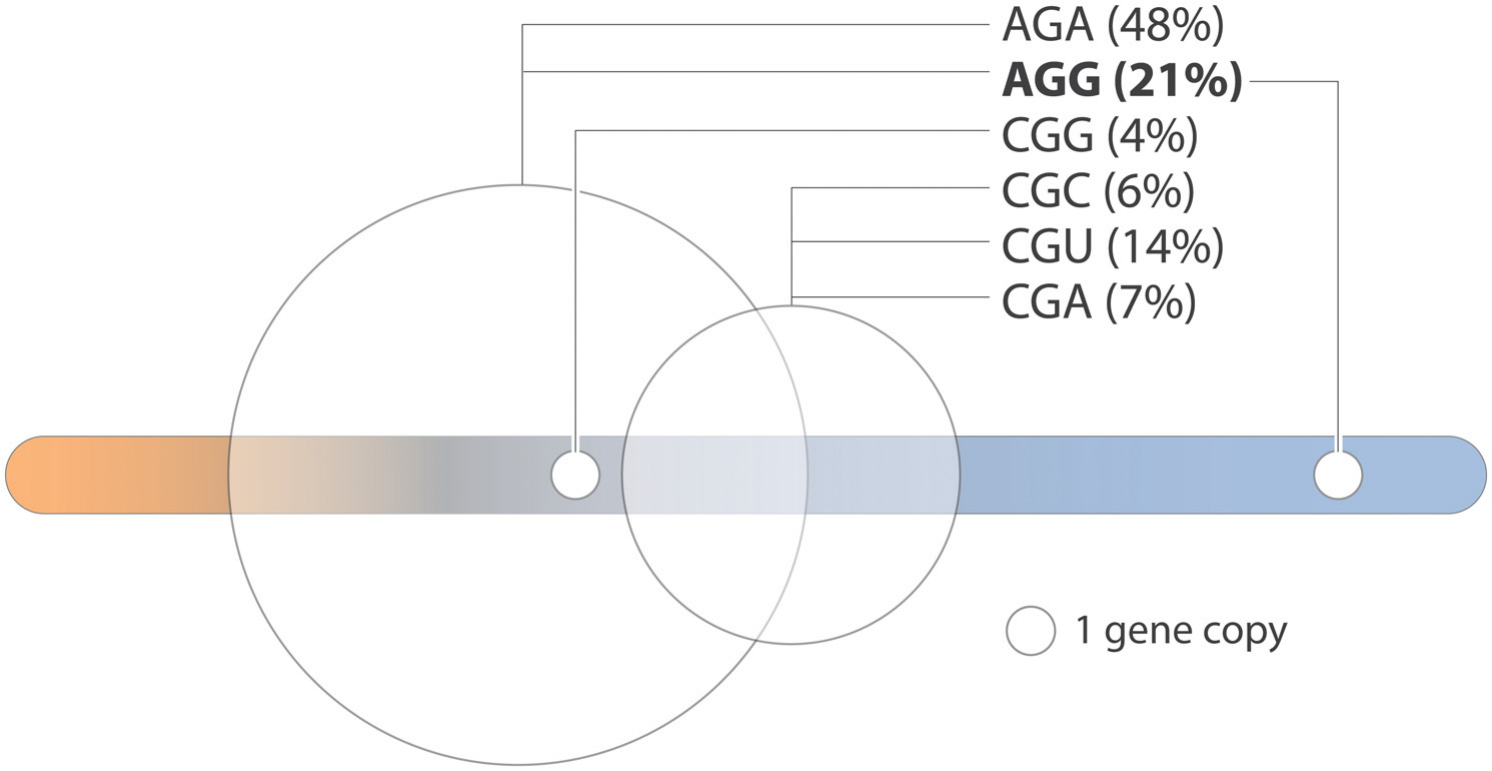
Yeast tRNA Arg and their decoding capacity. The figure describes the position on the affinity continuum for yArgRS as well as the arginine codons translated by the four tRNAs, Arg (UCU), (CCG), (ACG) and (CCU). A circle of proportional diameter represents the tRNA gene copy number. tRNA Arg (UCU) and (CCU) both translate AGG codons.

In *S*. *cerevisiae*, 4 tRNA Arg isoacceptors decode 6 arginine codons. These 4 species are scattered on the continuum of apparent affinities for yArgRS (Fig 8). tRNA Arg (UCU) has the highest apparent affinity; it is encoded by 11 gene copies (the highest copy number) and decodes 69% of arginine codons. Interestingly, tRNA Arg (CCU) that has redundant decoding capacity with tRNA Arg (UCU) has significantly poorer affinity for yArgRS. The latter is also encoded by single gene, which suggests a low intracellular abundance.

## Discussion

This work describes a new technical and conceptual approach, to address the specificity of RNA binding proteins such as aminoacyl-tRNA synthetases. We named this approach MIST for Microarray Identification of Shifted tRNA. Although MIST is a non-cellular assay, based on purified molecular partners, it integrates essential *in vivo* parameters such as tRNA stoichiometry as well as the presence of post-transcriptional modifications. More importantly, MIST fully incorporates the notion of tRNA competition.

tRNA microarrays were originally conceptualized and implemented in the laboratory of Dr. Tao Pan [26]. They have since become invaluable tools to study tRNA expression in healthy and diseased tissues [28, 32] or tRNA misacylation under stress conditions [33]. MIST constitutes the latest addition to the ever-growing tRNA microarray toolbox. It is based on a combination of gel retardation assays and tRNA microarrays. MIST decouples tRNA-AARS complex formation from tRNA aminoacylation to expose the hidden tRNA binding specificity of AARSs.

This work reveals new trends in tRNA-AARS complex formation:

1. *tRNAs are discriminated at the binding step*. yArgRS in submicromolar concentrations binds tRNAs with a wide range of apparent affinities. In addition, yArgRS preferentially binds to non-cognate tRNAs. Indeed, class 2 tRNA Ser and Leu significantly outcompete tRNA Arg for the binding to yArgRS under such conditions.
2. *Arginine identity is not required for tRNA binding only*. Top efficient binders, tRNA Ser and Leu lack arginine determinants supporting the notion that identity elements have been primarily selected throughout evolution for catalytic discrimination purposes.
3. *tRNA size is a contributing factor*. The top 6 efficient binders are all class 2 tRNAs suggesting that long variable arms are strong binding attributes. yArgRS displays an extensive surface area of compatible electrostatic potential dedicated for the binding of tRNAs [16]. The outline of this area matches the footprint of tRNA Arg on the enzyme implying a uniform binding mode for all interacting tRNAs including non-cognate species. According to this model, all tRNAs approach yArgRS by the minor groove of their acceptor stem exposing their variable loop to the solvent. As a consequence long variable arms of class 2 tRNAs are unlikely to clash with ArgRS but instead may provide the complex with additional binding energy. The superimposition of yArgRS on LeuRS/tRNA Leu complex reveals potential interactions with the long variable arm of tRNA Leu and the module named SC-fold [34]. This module follows the KMSKS loop in the active site of yArgRS. In addition, crystal structures and NMR spectroscopy have revealed extensive structural changes both on tRNA and ArgRS upon complex formation suggesting that class 2 tRNAs are prone to take full advantage of induced conformational changes.
4. *Accurate tRNA aminoacylation compensates for apparent lack of discrimination*. The most efficient binder, tRNA Leu (UAA), is not aminoacylated by yArgRS *in vivo* supporting the concept that catalytic discrimination constitutes the principal filter against misaminoacylation. In this regard, yArgRS appears to be particularly well prepared against fortuitous misaminoacylation due to the active participation of the complexed tRNA in the activation of arginine [4].
5. *yArgRS does not bind cognate tRNA Arg with the highest affinity*. yArgRS-tRNA complexes are stronger in the presence of tRNA Leu and Ser than with cognate tRNA Arg. In that respect, non-cognate tRNA Leu and Ser act as potential competitive inhibitors, which may sound counterproductive at first glance. Alternatively, a tight binding of non-cognate tRNA supports a fast turnover by facilitating the ejection of cognate tRNA after aminoacylation occurred.

This work raises questions:

### Is S. cerevisiae on the verge of loosing one tRNA Arg gene?

tRNA Arg (CCU) has a very low apparent affinity for yArgRS. Interestingly, this tRNA is encoded by a single copy gene and is functionally redundant for the translation of AGG codons with tRNA Arg (UCU) encoded by 11 genes (Fig 7). In a reasonable evolutionary scenario tRNA Arg (CCU) gene could be inactivated or directly reassigned by a single nucleotide substitution with no apparent impact on protein biosynthesis. Substitutions such as C35A, G or U would instantly inactivate the tRNA for aminoacylation and generate potential identity elements for Ala, Glu and GlyRSs respectively. Alternatively, strong evidence suggests that tRNA Arg (CCU) could actually be an original tRNA Asp acquired through genetic drifting after gene duplication and anticodon reassignment [35]. The low affinity observed in contemporary yeast could reflect the lag needed to reach optimal activity.

### What are the potential physiological implications?

Based on their mode of binding to the tRNA acceptor stem, Class I and class II AARSs have been subdivided into three subclasses. *In silico*, AARSs of opposite subclasses can bind simultaneously to the same tRNA with minimal steric clash [36]. Previous studies have pointed to the functional interrelationship between subclass Ia ArgRS and SerRS. In archaea, ArgRS assists SerRS in the serylation of tRNA Ser under extreme environmental conditions [37]. Both enzymes bind together to the large subunit of the ribosome to channel exiting tRNA towards aminoacylation [38]. The observation that yArgRS has a strong affinity for tRNA Ser suggests a similar connection between these two aminoacylation systems in *S*. *cerevisiae*. In yeast, ArgRS could act as a coenzyme by conveying tRNA Ser onto SerRS. Conversely, the previous observation that La protein Lhp1p assists yArgRS in aminoacylating tRNA Arg *in vivo* [39], suggests that yArgRs, in turn, needs coenzymes to aminoacylate low affinity isoacceptors.

### Do the trends observed for yArgRS apply to all AARSs?

tRNA and AARS expressions are regulated *in vivo* and their cellular concentrations can vary to a great extent [40, 41]. Because some AARSs have a great affinity for non-cognate tRNAs, an increased expression in the corresponding enzyme could sequester tRNA species away from their cognate AARSs and consequently slow down protein synthesis or even change global aminoacylation patterns by promoting misacylation. A methodical assessment of all yeast AARSs and their binding specificities would bring invaluable insights in the dynamics of the translation apparatus and the extent of its intermolecular network. With the emergence of systematic tools such as MIST, the achievement of a task of this magnitude appears finally realistic.

## Supporting Information

S1 File Probe sequences and microarray layout. (XLS)

S2 File Raw and processed tRNA microarray data. (XLSX)

S3 File Yeast tRNA sequences and gene copy number. (XLSX)

S1 Fig Influence of small substrate binding on tRNA-enzyme formation. (TIF)

S2 Fig Relative radioactive intensity of yeast total tRNAs hybridized to the array. (TIF)
