# Supplementary figures and images for: MIST, a Novel Approach to Reveal Hidden Substrate Specificity in Aminoacyl-tRNA Synthetases
Source: PLoS One. 2015 Jun 11;10(6):e0130042. doi: 10.1371/journal.pone.0130042 (PMC4465971; doi:10.1371/journal.pone.0130042)

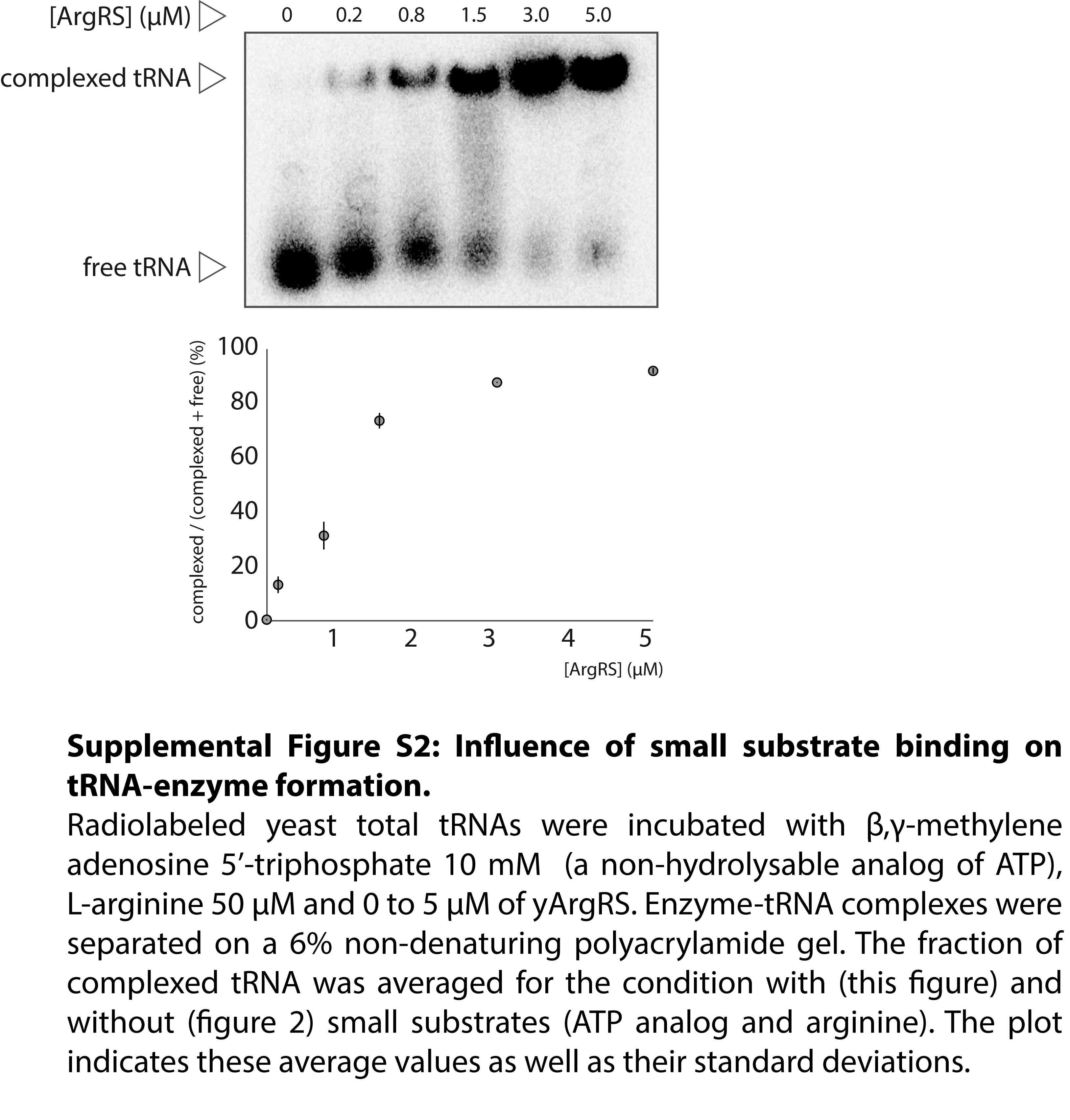

Supplement: S1 Fig — (TIF) [file pone.0130042.s004.tif]

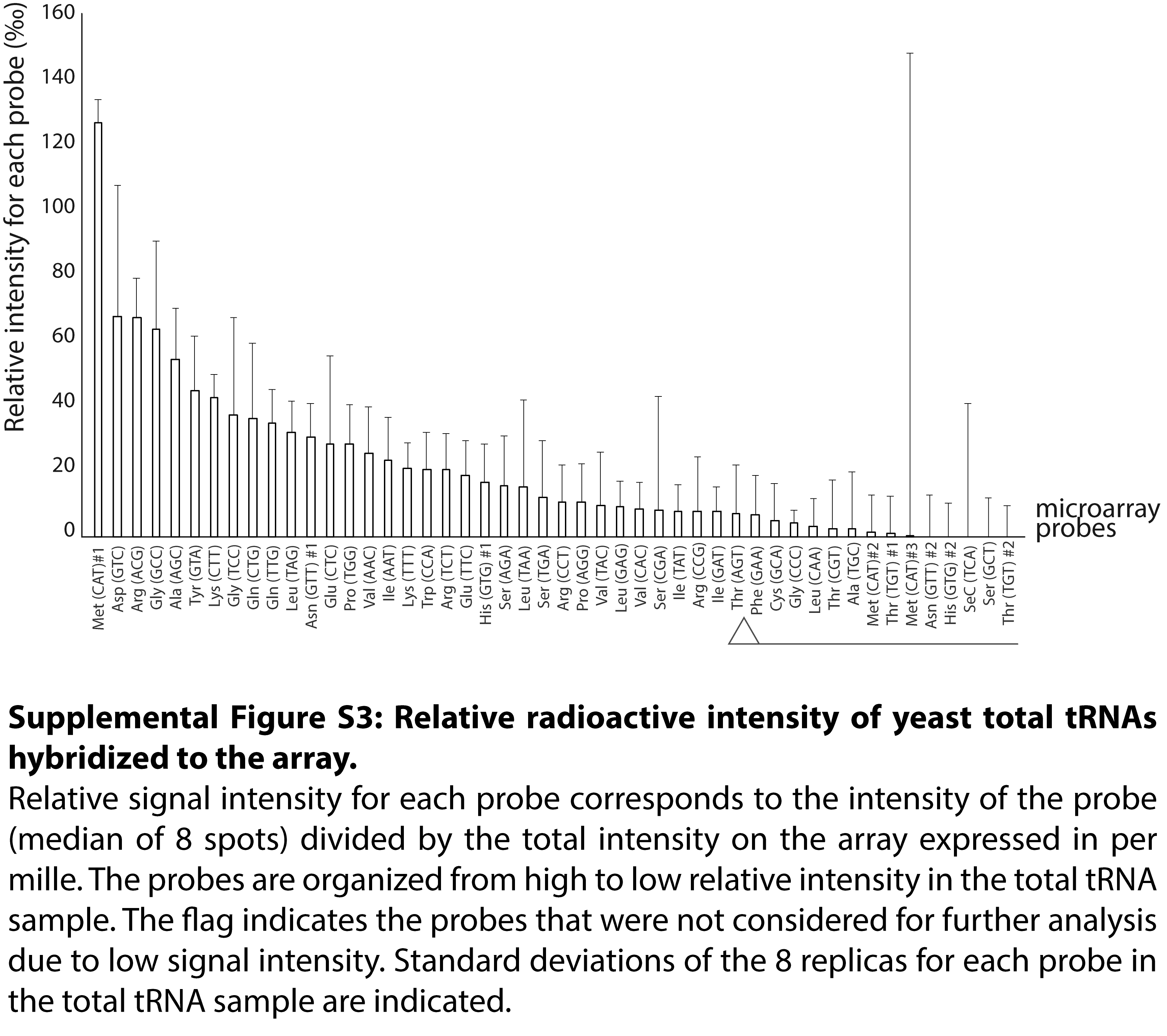

Supplement: S2 Fig — (TIF) [file pone.0130042.s005.tif]
